# Reducing Medication Problems among Minority Individuals with Low Socioeconomic Status through Pharmacist Home Visits

**DOI:** 10.3390/ijerph19074234

**Published:** 2022-04-01

**Authors:** Ya-hui Liang, Kai-Hsun Wang, Hung-Meng Huang, Ben-Chang Shia, Shang-Yih Chan, Chieh-Wen Ho, Chih-Kuang Liu, Mingchih Chen

**Affiliations:** 1 Graduate Institute of Business Administration, Fu Jen Catholic University, New Taipei City 242062, Taiwan; a2717@tpech.gov.tw (Y.-h.L.); king3300258@gmail.com (K.-H.W.); 025674@mail.fju.edu.tw (B.-C.S.); 2 Taipei City Hospital Heping Fuyou Branch, Taipei 100058, Taiwan; 3 University of Taipei, Taipei 111036, Taiwan; dad89@tpech.gov.tw; 4 Artificial Intelligence Development Center, Fu Jen Catholic University, New Taipei City 242062, Taiwan; cwho1220@gmail.com; 5 Department of Otolaryngology, School of Medicine, College of Medicine, Taipei Medical University, Taipei 110301, Taiwan; dae91@tpech.gov.tw; 6 Department of Otolaryngology, Taipei City Hospital, Taipei 10341, Taiwan; 7 Department of Health Care Management, National Taipei University of Nursing and Health Sciences, Taipei 10845, Taiwan; 8 Department of Internal Medicine, Taipei City Hospital Yangming Branch, Taipei 111024, Taiwan; 9 Department of Life Science, National Taiwan University, Taipei 10617, Taiwan; 10 MS Program of Long Term Care, Fu-Jen Catholic University, New Taipei City 242062, Taiwan; 11 Department of Urology, Fu-Jen Catholic University Hospital, New Taipei City 24352, Taiwan

**Keywords:** Home visit, Pharmacist, Medication problem, Low socioeconomic

## Abstract

Introduction: In this study, pharmacists conducted home visits for individuals of medically underserved populations in Taiwan (i.e., socioeconomically disadvantaged individuals, middle-aged or older adults, and individuals living alone, with dementia, or with disabilities) to understand their medication habits. We quantified medication problems among various groups and investigated whether the pharmacist home visits helped to reduce the medication problems. Materials and Methods: From April 2016 to March 2019, pharmacists visited the homes of the aforementioned medically underserved individuals in Taipei to evaluate their drug-related problems and medication problems. Age, living alone, diagnoses of dementia or disabilities, and socioeconomic disadvantages contributed significantly to inadequate disease and medical treatment knowledge and self-care skills as well as lifestyle inappropriateness among patients. The patients who were living alone and socioeconomically disadvantaged stored their drugs in inappropriate environments. Results: After the pharmacists visited the patients’ homes twice, the patients improved considerably in their disease and medical treatment knowledge, self-care skills, and lifestyles (*p* < 0.001). Problems related to the uninstructed reduction or discontinuation of drug use (*p* < 0.05) and use of expired drugs (*p* < 0.001) were also mitigated substantially. Discussion and conclusion: Through the home visits, the pharmacists came to fully understand the medicine (including Chinese medicine) and health food usage behaviors of the patients and their lifestyles, enabling them to provide thorough health education. After the pharmacists’ home visits, the patients’ drug-related problems were mitigated, and their knowledge of diseases, drug compliance, and drug storage methods and environments improved, reducing drug waste. Our findings can help policymakers address the medication problems of various medically underserved groups, thereby improving the utilization of limited medical resources.

## 1. Introduction

In 1993, Taiwan became an aging society, and in 2018, it became an aged society (i.e., one with more than 14% of the population aged ≥65 years). It is expected to become a superaged society by 2025, and the pace of its transition to a superaged society is more rapid than those of other countries [1]. According to the 2021 statistics of Taiwan’s Ministry of the Interior, older adults aged ≥65 years account for 16.15% of Taiwan’s population. With 496,991 (19.13%) of its residents being older adults, Taipei City has the highest percentage of older adults among Taiwan’s six special municipalities and the third-highest among Taiwan’s major administrative divisions. Rapid population aging poses various problems to society; other than medical needs, older adults’ financial, daily care, residential, spiritual, and self-realization needs must also be satisfied. The impact of population aging on medical, sociopolitical, and economic systems poses tremendous challenges for relevant authorities.

Taiwan has a compulsory National Health Insurance system. In 2019, the medical expenses for inpatient and outpatient services (including emergency services) amounted to 722.3 billion points (dollar), of which 38.4% was associated with services for older adults [2]. The high accessibility of medical services in Taiwan and low fees for services (approximately $3.3–$13.7 per clinic visit) have given Taiwanese residents the tendency to seek as much medical help as possible; this has led to a behavior known as hospital shopping. In the previous decade, the average Taiwanese resident made 17 clinic visits per year [2], resulting in the dispensing and consumption of numerous drugs. Polypharmacy and drug interactions are closely associated with adverse drug reactions, which are particularly prevalent among older adults, who tend to use drugs inappropriately [3,4]. Concerns such as repeat clinic visits, duplicate prescriptions, and complicated patient health demands must be addressed to avoid overburdening Taiwan’s National Health Insurance system [5,6].

Furthermore, because of the COVID-19 pandemic, the government has restricted people from visiting hospitals, and thus, home-care visits have become essential for accessing medical resources. Given limited resources, home visits need to be effective.

In Taiwan, low- or moderate-income families are those in which each member’s per capita share of the total household income is no higher than 1.5 times the minimal monthly living expenses as determined by region or those with properties valued below the maximal amount for a low- or moderate-income household in the region. In 2021, the minimal monthly living expense for an individual in Taipei was $631. For a family to be considered to have a low or moderate income, the value of assets per person must not exceed $5357, and the total property value must not exceed $264,285 [7]. Socioeconomically disadvantaged individuals include those with disabilities, in long-term unemployment, or from single-parent families as well as disadvantaged women (e.g., women dependent on financial supporters, indigenous women, women reemployed after long unemployment, women who have experienced domestic violence or sexual assault, women without education or employment, adolescent girls from remote areas or crisis-stricken areas, and women from economically disadvantaged households).

Pharmacists in medical institutions in Taiwan typically provide pharmaceutical services, such as dispensing drugs according to prescriptions. However, generally, insufficient time is available for medication consultations, resulting in many drug-related problems (DRPs). The Taiwan Quality Improvement of Pharmaceutical Affairs Association surveyed 20 medical centers and revealed that the average medication consultation time was only 35 s [8]. Poor communication and insufficient information are often the reasons for DRPs. As many as 10–30% of inpatients are hospitalized due to DRPs, such as an incorrect drug prescription, an incorrect dosage, an inadequate prescription, and poor adherence; older adults are especially susceptible to DRPs because of their general decline in organ functions [9]. However, such DRPs can be prevented [10,11]. The prevalence of hospitalization owing to adverse drug reaction increases with age. Numerous studies have demonstrated that the intervention of medical teams can effectively reduce the number of clinic visits, duration of hospitalizations, number of recurrent emergency room visits, and even the mortality rate of patients. Moreover, pharmacists’ intervention can reduce the number of drugs prescribed and, thus, overall medical expenditures [12,13,14].

In Taiwan, home visits for patients are uncommon. In this study, we investigated the medication problems of patients with low socioeconomic status and whether home visits could improve their medication habits. 

## 2. Materials and Methods

### 2.1. Study Population

In this study, we focused on minority patients with low socioeconomic status. From April 2016 to March 2019, middle-aged or older adults who were living alone, had dementia, had disabilities, or were economically disadvantaged and who agreed to a home visit were enrolled as participants. Patients’ demographic data (i.e., sex, gender, education level, and mobility) were collected. Because the study involved a public hospital, the participants of the home visit program were former inpatients and patients who were visited at the behest of the village chief.

### 2.2. Assessments

Assessments of patients’ current medical treatment, economic status, and living conditions were conducted, and the pharmacists were asked to provide the appropriate pharmaceutical services. Patients were invited to join the project voluntarily, and their written consent was obtained during the first home visit. The pharmacists searched for medicines (including Chinese herbal medicines) and health foods in the patients’ homes and inquired about their medication indications, dosage, intake frequency, and treatment duration as well as about adverse reactions and other problems related to their prescriptions. After the pharmacists interviewed the patients, they completed an evaluation form (comprising prescription assessment items, suggestions, and medication guidance) to report the patients’ conditions.

Items related to prescription assessment were designed to evaluate the following: whether a prescription was problematic, whether patients had insufficient knowledge of their disease and medical treatment, whether patients had insufficient knowledge of drug use, patients’ medication adherence, drug storage problems, and drug expiration problems (Table 1).

**Figure 1:**
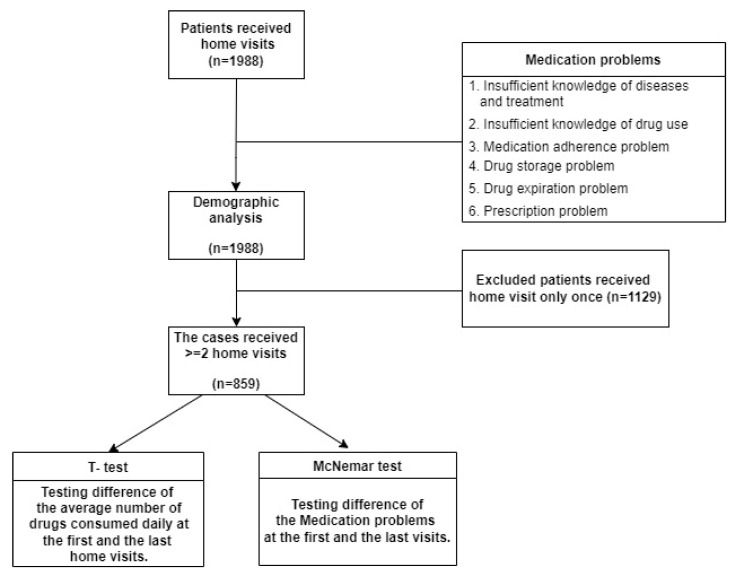
Flowchart for assessment of changes in medication problems between the first to the last home visit.

The research flowchart is presented in Figure 1. In all, 1988 people received home visits (Table 2). Of them, 859 received multiple home visits.

The pharmacists also provided medication-related consultations and helped to recycle expired drugs. The pharmacists provided medication consultation and education and life- and drug-related suggestions, the physicians conducted medical evaluations, the social workers helped to address the patients’ daily life concerns and social welfare, and the dieticians focused on the patients’ nutrition. The project was patient-centered and provided patients with personalized medical advice.

The key criteria that determined whether a patient required a second visit were problematic prescriptions, insufficient medical knowledge, or an excessive amount of unused drugs. For patients who received two or more visits, the evaluation forms for the first and final visits were compared.

### 2.3. Statistical Analysis

A chi-square test was used to analyze the participants’ medication problems. For patients who received multiple home visits, the McNemar test was used to compare the results of the first and final visits and thereby evaluate the effectiveness of the visits, and the t-test was used to compare the number of drugs taken each day between the first and last visits. The collated data are presented in a 2 × 2 table. 

## 3. Results

Between April 2016 and March 2019, 1988 patients received home visits. Table 2 presents the patients’ demographic information. Of the 1988 patients, 975 (49%) were men, 1013 (51%) were women, 1704 (85.8%) were aged ≥60 years, and 1280 (64.4%) had not received any formal education (*n* = 750) or had received only elementary school education (*n* = 530).

Table 3 indicates that middle to old age (Yes or No), living alone (Yes or No), disadvantaged status (Yes or No), disabilities (Yes or No), and dementia (Yes or No) contributed to significant differences in whether patients’ knowledge was sufficient regarding treatment, their self-care skills were sufficient, and their lifestyle was appropriate. Table 4 presents all significant differences (*p* < 0.01). 

To determine the effectiveness of home visits, we analyzed only those patients who received multiple home visits (*n* = 859; Table 4). Pharmacist intervention significantly influenced the problems relating to drug indications, therapeutic duplication, and an inadequate treatment period. Patients’ insufficient knowledge related to disease and treatment, self-care skills, inadequate lifestyle, and insufficient knowledge of medication were significantly improved after pharmacist intervention. In terms of medication adherence, the patients only showed improvements in deliberately reducing the number of medications because of concerns regarding taking too many drugs and in medication stoppage because of concerns regarding side effects; no improvement was observed in forgetting to take medication, taking the wrong medication, or stopping a medication because of poor taste. The proportion of patients exhibiting inappropriate storage environment/location/method declined from 13% to 10% (*p* = 0.0029). Significant improvements were also observed in behaviors relating to problems of drug expiration: The proportion of expired medicines not discarded that had been discarded declined from 9.7% to 5.4%, and the proportion of remaining medicine with an unknown expiry date decreased from 7.3% to 4.2% (both *p* < 0.0001; Table 4).

The average number of drugs consumed daily decreased from 8.8 to 8.5 (*p* = 0.11; Table 5).

## 4. Discussion

Improving the health and medication management of patients with complicated needs, such as older adults, is challenging for health authorities. In Taiwan, pharmacists do not receive a fixed payment for providing pharmaceutical services through home visits. Although short-term home visit programs involving pharmacists’ services have been considerably successful in numerous countries [15,16], such programs require substantial medical resources. Therefore, a comprehensive plan must be established to achieve considerable improvements throughout the medical industry.

A qualitative study on the topics covered during community pharmacist home visits following discharge observed that patients exhibited problems related to medication knowledge, use, and storage [17], which is consistent with our findings. An essential strength of this study was its large sample size, as this is the first study to quantitatively compare the effectiveness of first and second home visits. Previous studies have used only in-depth interviews to survey drug problems during home visit interventions, and such interventions have improved patients’ self-care skills, thus enabling them to make lifestyle changes and consequently improve their health [18]. Patients with low education levels might have difficulty presenting questions relevant to medication [19]. In this study, the percentage of patients who had not received formal education was as high as 37.7%. Given the patients’ possible difficulty in appropriately expressing their questions, a standard questionnaire was used by the pharmacists during the home visits to assist them in quickly identifying DRPs. Moreover, home visits allowed the pharmacists to observe the patients’ living conditions and identify possible problems. In addition, caregivers should also be included in health education and assessment to optimize the improvement in patient health [20]. 

In patient-centered care, communication skills are critical to familiarizing patients with their medication and encouraging them to follow medication instructions [21]. In a medical institution, pharmacists are only in charge of dispensing drugs according to prescriptions and are rarely required to identify patients’ DRPs by observing patient habits. Therefore, pharmacists should be trained before they make home visits. During a home visit, a pharmacist must establish rapport with the patient so that the patient will be comfortable presenting their problems, show the pharmacist all the drugs they are taking, including traditional Chinese and Western medicines as well as health foods, and allow the home visit team to examine their refrigerator and rice cooker to determine the patient’s actual dietary habits. This is particularly important because, during clinic visits, patients with diabetes may often claim that they do not drink sweetened beverages. However, the home visit team may find a refrigerator stocked with such beverages in the patient’s house. Examining the refrigerator also provides the team insight into a patient’s drug storage habits—many patients habitually store their drugs in the refrigerator. A pharmacist must be trained to make the patient feel comfortable through a combination of professional medication consultation and small talk so that the patient openly reveals their real problems.

The pharmacist can also serve as the mediator between the patient and other members of the home visit team. Side effects of medicines, poor financial condition, religious beliefs, mental illness, and impaired memory can promote poor medication adherence [22]. A pharmacist can explore these factors to determine whether poor adherence results from a complicated medication schedule, fear of side effects, poor taste, or poor memory. Medication adherence is critical to a patient’s treatment progress [23]. Individuals who live alone or are economically disadvantaged often forget to take medication, possibly because they have no family to remind them or are busy with work, respectively. Therefore, a smart reminder method is required for patients in these categories.

In Taiwan, the ease of obtaining medical services has resulted in an annual average of 17 clinic visits per person [24]. This not only highlights a lack of coordination in medical services but also how susceptible the system is to abuse and wasted medical resources. This study investigated the effectiveness of home visit medical teams, which comprised at least one pharmacist, in patient-centered health education and medical integration for resolving the problem of patients visiting various clinics and taking a number of drugs for multiple chronic diseases. Home visits allow pharmacists to provide face-to-face medication consultation, prescription-related advice, and guidance on leading a healthy lifestyle. The results indicated that the number of pills a patient took per day declined by 0.3, which was nonsignificant and lower than that observed in the CPHV program [12]. This result may be attributable to the inclusion criteria, which were not designed specifically to obtain information regarding polypharmacy.

A strength of this study is the large sample size. The data were collected over three years and in a single medical institution through a structured questionnaire, which improved the consistency of pharmacists’ home visit results. All the participating pharmacists worked in the same medical institution, resided in the same county, and received the same home visit training. Although their years of service varied, an effort was made to reduce their differences to the largest extent possible. Because the medical institution selected in the study was a public hospital, the participants of the home visit program included inpatients and patients who were visited on the referral of village chiefs. This promoted mutual understanding between communities and hospitals.

This study has some limitations. One limitation was budgetary restrictions. On average, a team could only complete four visits in one afternoon; the visits were time-consuming [25], and the costs involved in terms of human resources and time consumption were high. Another limitation was the lack of follow-up management of the patients. Despite efforts to minimize pharmacists’ before-visit differences, few conducted follow-ups.

## 5. Conclusions

In this study, we discovered that home visits were effective in reducing the medication problems of low socioeconomic minorities. Future studies should try combining home visits with telemedicine to more effectively alleviate medication problems among such individuals. This study was initiated from the home visit program for one public hospital which is one of the research limitations. It is suggested that a larger data study can be carried out in the future.

The home visits enabled the pharmacists to fully understand the medications (including Chinese herbal medicines) and dietary behaviors of the patients. This enabled them to provide thorough health education to the patients. After the pharmacists’ home visits, the patients’ DRPs, including repeat medication, were mitigated; their knowledge of diseases, drug compliance, and drug storage methods and environments improved, thereby reducing drug waste and improving patient drug safety.

Our findings may guide policymakers in establishing subgroup-specific interventions to address various medication problems. The resulting policies may help to improve the utilization of limited medical resources, including time, labor, and money.

## Figures and Tables

**Table 1:** Assessment definition.

Items	Definition
1.Insufficient knowledge of disease and treatment	1.1. Insufficient knowledge/erroneous understanding of disease and personal health condition
	1.2. Inadequate self-care skills
	1.3. Inappropriate lifestyle (diet/nutrition/exercise)
	1.4. Insufficient knowledge/erroneous understanding of health promotion/disease prevention
2.Insufficient knowledge of drug use	2.1. Patient does not understand medication indications/usage/contraindications
	2.2. Patient does not understand precautions/side effects
	2.3. Patient does not understand dosage
	2.4. Patient does not understand correct medication usage
	2.5. Patient does not understand correct storage method
	2.6. Patient is unfamiliar with information labeled on medicine bag
3.Medication adherence problem	3.1. Patient often forgets to take medicine
	3.2. Patient sometimes forgets to take medicine
	3.3. Patient reduces dosage because of concerns regarding adverse effects of excessive medication use
	3.4. Patient discontinues medication use because of side effects
	3.5. Patient reduces/discontinues medication use because of the unpleasant taste of medicine
4.Drug storage problem	4.1. Inappropriate storage environment/location/method
	4.2. Inappropriate storage temperature
	4.3. Medicine bag is missing/name of the medicine is unclear
5.Drug expiration problem	5.1. Patient continues to take expired medicine
	5.2. Patient does not dispose of expired medicine
	5.3. Patient has unused medicine with an unknown expiry date
	5.4. Patient does not routinely check the expiry dates of medicines
6.Prescription problem	6.1. Indications
	6.2. Duplicate medications
	6.3. Interaction
	6.4. Overdose
	6.5. Under dose
	6.6. Inaccurate dosage
	6.7. Frequency problem
	6.8. Inappropriate medication for the treatment period
	6.9. Monitor lab data/TDM (therapeutic drug monitoring)
	6.10. ADR (adverse drug reaction)

**Table 2:** Participants’ demographic characteristics.

Demographic Variables		
	N	%
N	1988	100
Gender		
man	975	49.0
female	1013	51.0
Age group (years)		
<20	6	0.3
21–30	3	0.2
31–40	32	1.6
41–50	89	4.5
51–60	154	7.7
61–70	259	13.0
71–80	355	17.9
81–90	665	33.5
>90	425	21.4
Education level		
no education	750	37.7
elementary	530	26.7
secondary	263	13.2
senior high school	280	14.1
university	165	8.3
Activity		
function independent	1016	51.1
use assistive devices	320	16.1
use a wheelchair	279	14.0
long-term bed rest	373	18.8

**Table 3:** Patients’ medication problems.

		Non-Middle-Aged/Middle-Aged	Not Living Alone/Living Alone	Non-Disadvantaged/Disadvantaged Status	Have Daily Physical Function/Loss of Daily Physical Function	Non-Dementia/Dementia
1. Insufficient knowledge of diseases and treatment	1.1 Insufficient knowledge/error of illness and medical treatment	0.0124	<0.0001 **	<0.0001 **	0.2598	0.0035 *
	1.2 Insufficient self-care skills	<0.0001 **	<0.0001 **	<0.0001 **	0.0148	0.0004 **
	1.3 Inappropriate lifestyle (diet/nutrition/exercise)	<0.0001 **	<0.0001 **	<0.0001 **	0.0282	0.0003 **
	1.4 Inadequate awareness/errors in health promotion/disease prevention	0.0867	0.0022 *	0.0059 *	0.0017 *	0.0209
2. Insufficient knowledge of drug use	2.1 Do not understand the indications/uses of the drug	0.2396	0.2165	<0.0001 **	0.172	0.0107
	2.2 Do not understand the precautions/side effects/contraindications of drug use	0.037	0.2562	0.0002 **	0.2504	0.6017
	2.3 Do not know the usage and dosage of drugs	0.2932	0.1753	0.0004 **	0.3443	0.0009 **
	2.4 Don’t understand how the drug is used	0.2299	0.4017	0.4529	0.1525	0.0007 **
	2.5 Don’t know the correct way to store the medicine	0.1641	0.2874	0.1464	0.3771	0.4712
	2.6 Not familiar with the labeling information of the medicine bag	0.3252	0.0481	0.1695	0.986	0.0673
3. Medication adherence problem	3.1 Often forget to take medicine	0.3924	<0.0001 **	<0.0001 **	0.2155	0.1547
	3.2 Occasionally take the wrong medicine	0.6962	0.2523	0.0768	0.3123	0.2275
	3.3 Feel that taking too much medicine is not good to reduce the amount of medicine	0.4948	0.4069	0.1643	0.2212	0.5779
	3.4 Discontinue the medication by yourself because of the side effects of the medication	0.1241	0.445	0.354	0.1483	0.0732
	3.5 Feel that the medicine tastes bad, reduce the dosage or stop the medicine by yourself	0.6054	0.1245	0.9354	0.2559	0.8221
4.Drug storage problem	4.1 Inappropriate storage environment/location/method	0.0022 *	0.0031 *	<0.0001 **	0.1204	0.1656
	4.2 Improper storage temperature	0.1807	0.3846	0.7477	0.0153	0.1106
	4.3 The medicine bag is not kept or the name of the medicine is unclear	0.0275	0.0714	0.0547	0.1528	0.115
5. Drug expiration problem	5.1 Continue to take expired drugs	0.0133	0.0001 **	0.121	0.2654	0.1128
	5.2 Expired medicines are not discarded	0.1923	0.7748	0.7787	0.0569	0.9934
	5.3 Remaining medicine with the unknown expiry date	0.4439	0.6784	0.3915	0.7389	0.0942
	5.4 No habit of regularly checking the expiration date of drugs	<0.0001 **	0.0873	0.157	0.7711	0.2279

If there are more than 80% of the cells, the number of samples is less than or equal to 5, the chi-square test will not be displayed in the table. * Significant at *p* < 0.01. ** Significant at *p* < 0.001.

**Table 4:** Medication problems at the first and last home visits.

		First Visit		Last Visit		McNemar Test
		N	%	N	%	*p*-Value
1. Insufficient knowledge of disease and treatment	1.1 Insufficient knowledge/error of illness and medical treatment	291	33.9	226	26.3	<0.0001 *
	1.2 Insufficient self-care skills	336	39.1	269	31.3	<0.0001 *
	1.3 Inappropriate lifestyle (diet/nutrition/exercise)	248	28.9	206	24.0	<0.0001 *
	1.4 Inadequate awareness/errors in health promotion/disease prevention	381	44.4	346	40.3	00.0161
2. Insufficient knowledge of drug use	2.1 Do not understand the indications/uses of the drug	284	33.1	203	23.6	<0.0001 *
	2.2 Do not understand the precautions/side effects/contraindications of drug use	273	31.8	202	23.5	<0.0001 *
	2.3 Do not know the usage and dosage of drugs	227	26.4	152	17.7	<0.0001 *
	2.4 Don’t understand how the drug is used	167	19.4	120	14.0	<0.0001 *
	2.5 Don’t know the correct way to store the medicine	66	7.7	49	5.7	0.0063 *
	2.6 Not familiar with the labeling information of the medicine bag	86	10.0	63	7.3	0.0026 *
3. Medication adherence problem	3.1 Often forget to take medicine	87	10.1	76	8.8	0.123
	3.2 Occasionally take the wrong medicine	58	6.8	47	5.5	0.055
	3.3 Feel that taking too much medicine is not good to reduce the amount of medicine	80	9.3	59	6.9	0.0069 *
	3.4 Discontinue the medication by yourself because of the side effects of the medication	57	6.6	43	5.0	0.016
	3.5 Feel that the medicine tastes bad, reduce the dosage or stop the medicine by yourself	18	2.1	17	2.0	0.7815
4. Drug storage problem	4.1 Inappropriate storage environment/location/method	112	13.0	87	10.1	0.0029 *
	4.2 Improper storage temperature	35	4.1	28	3.3	0.1439
	4.3 The medicine bag is not kept or the name of the medicine is unclear	74	8.6	62	7.2	0.1083
5. Drug expiration problem	5.1 Continue to take expired drugs	41	4.8	32	3.7	0.049
	5.2 Expired medicines are not discarded	83	9.7	46	5.4	<0.0001 *
	5.3 Remaining medicine with the unknown expiry date	63	7.3	36	4.2	<0.0001 *
	5.4 No habit of regularly checking the expiration date of drugs	140	16.3	109	12.7	0.0031 *
6. Prescription problem	6.1 Indication	72	8.4	46	5.4	0.0001 *
	6.2 Duplicate medication	21	2.4	11	1.3	0.0037 *
	6.3 Interaction	13	1.5	7	0.8	0.0827
	6.4 Overdose	19	2.2	12	1.4	0.0890
	6.5 Under dose	8	0.9	4	0.5	0.1568
	6.6 Inaccurate dosage	15	1.7	9	1.0	0.0334
	6.7 Frequency problem	40	4.7	31	3.6	0.1167
	6.8 Inappropriate medication for the treatment period	20	2.3	11	1.3	0.0197
	6.9 Monitor lab data/TDM (therapeutic drug monitoring)	95	11.1	68	7.9	0.0006 *

If more than 80% of the cells or the number of samples is ≤5, the chi-square test result is not displayed. * Significant at *p* < 0.01.

**Table 5:** Number of drugs taken per day at the first and last home visits.

	Variable	Average Value	Standard Deviation	Minimum	Max	*p*
First visit	Number of oral medications per day	8.8	6.1	0	36.5	0.1125
Last visit	Number of oral medications per day	8.5	5.8	0	35	

## Data Availability

Data available on request due to restrictions. The data presented in this study are available on request from the corresponding author. The data are not publicly available due to privacy and ethical.
